# Behavioral Priming: It's All in the Mind, but Whose Mind?

**DOI:** 10.1371/journal.pone.0029081

**Published:** 2012-01-18

**Authors:** Stéphane Doyen, Olivier Klein, Cora-Lise Pichon, Axel Cleeremans

**Affiliations:** 1 Consciousness, Cognition and Computation Group, Université Libre de Bruxelles, Brussels, Belgium; 2 Social Psychology Unit, Université Libre de Bruxelles, Brussels, Belgium; 3 Social and Developmental Psychology Department, University of Cambridge, Cambridge, United Kingdom; Kyushu University, Japan

## Abstract

The perspective that behavior is often driven by unconscious determinants has become widespread in social psychology. Bargh, Chen, and Burrows' (1996) famous study, in which participants unwittingly exposed to the stereotype of age walked slower when exiting the laboratory, was instrumental in defining this perspective. Here, we present two experiments aimed at replicating the original study. Despite the use of automated timing methods and a larger sample, our first experiment failed to show priming. Our second experiment was aimed at manipulating the beliefs of the experimenters: Half were led to think that participants would walk slower when primed congruently, and the other half was led to expect the opposite. Strikingly, we obtained a walking speed effect, but only when experimenters believed participants would indeed walk slower. This suggests that both priming and experimenters' expectations are instrumental in explaining the walking speed effect. Further, debriefing was suggestive of awareness of the primes. We conclude that unconscious behavioral priming is real, while real, involves mechanisms different from those typically assumed to cause the effect.

## Introduction

In their seminal series of experiments, Bargh Chen and Burrows [1] demonstrated that activating a trait construct such as “being old” is sufficient to elicit behavioral effects in the absence of awareness. Bargh et al.'s demonstration involved asking participants to indicate which word was the odd one out amongst an ensemble of scrambled words a number of which, when rearranged, form a sentence. Unbeknownst to participants, the word left out of the sentence was systematically related to the concept of “being old”. The beauty of the experiment lies in its unusual dependent measure: walking speed. Those participants who had been exposed to words related to old age walked slower when exiting the laboratory than the participants who had not been so exposed. Further, the effect was claimed to occur without awareness, as participants were found not having noticed the link between exposure and their behavior [1]. This striking finding, now widely cited, established that priming may occur automatically and influence behavior with little or no awareness. It subsequently generated considerable further research in social psychology [2], [3], [4], [5].

Here, we sought to replicate Bargh et al's [1] experiments. This was motivated by three main reasons. The first is simply that the finding, influential as it is, was only replicated twice so far, with neither replication being exact. The first replication [6] required participants to rate the walking speed of a character drawn on a sheet of paper after they had been primed with extreme exemplars associated to the concept of “speed” (e.g.: cheetah vs. turtle). The study found the expected priming effect. However, despite the fact that both this study and Bargh et al's share the same theoretical grounding, the latter only tells us of a bias in judgments of speed, as it did not require participants to actually perform any behavior. The second [7] replication aimed at refining Bargh et al's results by exploring the substantial variability exhibited by participants in their priming effect. The authors managed to replicate the results on walking speed, but the replication, like the original study, can be questioned based on imprecise timing methods (see below for a critical description).

Our second motivation was more conceptual. In social cognition, the assumption that high level semantic priming can occur automatically and outside of conscious awareness is almost taken for granted [8]. Yet, this assumption conflicts with evidences accumulated in cognitive neuroscience. For instance, several authors [9], [10], [11] suggest that two factors are necessary to produce the large patterns of neural activation in higher association cortices that are essential for semantic priming to occur: Top-down attention to the prime and bottom-up stimulus strength (e.g.: its saliency to the participants). Such a pattern of activation can be the signature of semantic processing of the prime [12] and is typically associated with conscious awareness [13]. In Bargh et al.'s experiments 2a and 2b, however, neither of these features were present. Thus, in view of the semantic priming literature, the salience of a concept such as “being old” seems too weak to automatically prime a behavior that is further only indirectly related to old age (i.e., through the concept of slowness), in the absence of any contextual cues relevant to this trait. In Bargh et al.'s account, the association between the categorical prime (old age) and walking speed (a behavior) is mediated by the automatic activation a stereotypical trait (slowness).

The absence of conscious awareness of the relation between the prime and the behavior is considered as evidence that this activation is indeed automatic.

Our third motivation is methodological. Some aspects of Bargh et al.'s [1] experiment's 2a and 2b remain unspecified yet impinge on the interpretation of the results. In particular, we see three potential challenges with the methods of the original study:

First, in the original study, participants' walking speed was measured by a confederate posted in the hallway adjacent to the experiment room. The confederate was unaware of whether a participant had been primed or not. The purpose of this setup was to ensure that the experiment was following a double-blind principle. However, no such precautionary measures are reported concerning the experimenter who administered the task to the participants. Numerous studies, however, have indicated that the experimenter's expectations can influence participants' behavior [14] even in the most controlled experimental environments [15]. In Bargh et al.'s study, the experimenter who administered the task could thus very well have been aware of whether the participant was in the prime condition or not and tune his or her behavior accordingly. This possibility was in fact confirmed informally in our own study, as we found that it was very easy, even unintentionally, to discover the condition in which a particular participant takes part by giving a simple glimpse to the priming material. Experimenters could thus unwittingly have communicated their expectations to participants [16] and influenced their walking speed. Thus, given the fact that subtle cues can influence our behavior [17], controlling for the experimenter's expectations appears to be essential.

Second, walking speed was measured using a manual stopwatch — a method that is prone to error and bias. Manual chronometry requires extra precautions [7] which appear to be absent from the original study.

Third, after the experiments, participants were debriefed using the contingency funnel procedure [18], [19] so as to assess, through increasingly specific questions, (1) whether they were aware of the purpose of the study and (2) whether they were aware of the fact that the words used in the scrambled sentences task were related to the concept of old age. Only 1 out of 19 participants [1] (p.237) were found to be aware of the influence of the primes, — a finding that formed the basis for the claim that the effect of stereotype activation on behavior is unconscious. However, it remains unclear exactly what participants claimed to be unaware of. As Nisbett and Wilson [20] famously pointed out, participants can remain (1) unaware of the stimulus, (2) unaware of their response, or (3) unaware of the fact that the stimulus importantly influenced the response. Thus, we set out to improve on the tests of awareness originally used by Bargh et al. [1] in hopes of better delineating exactly what people were aware of in this situation.

In the following, we report on two studies aimed specifically at replicating the original findings while improving on its design and exploring the extent to which the experimenter's own expectancies may influence the results. In Experiment 1, we sought to replicate the original study but used automated rather than manual chronometry. In Experiment 2, we directly manipulated experimenters' expectancies by making half of the experimenters think their participants would slow down after exposure and the other half think that their participants would speed up after exposure.

## Experiment 1

### Methods

#### Participants

120 undergraduate Belgian French speaking students (age range 20–34 years of age, average 21.9) took part in Experiment 1. Four experimenters were recruited for this experiment (age range 21–24 years of age, average 22.5). This experiment was a mandatory component of a practical course in psychology and was approved by the Ethics Committee of the Department of Psychological Sciences of the University of Brussels. Participants gave verbal informed consent to participate in this study, for which no particular written consent form was necessary, as they already had provided a general consent for the course to the Faculty of Psychology. This procedure was specifically approved by the Ethics Committee mentioned above.

#### Procedure and Design

Participants were told that they had to take part in a test of their French linguistic skills.

The design replicated Bargh et al.'s [1] study and took place in an empty hallway located on a vacant floor of a Université Libre de Bruxelles building. The hallway, a long featureless corridor, led to the experimental room. Two infrared sensors were hidden in the hallway, separated by the same distance as in the original study (9.75 m). The recording of the sensors signal was started before a participant arrived and was stopped upon full completion of all tasks. Participants were clearly directed to the end of the corridor to avoid any wandering and crossed each beam on each passage. There were two conditions defined by which version of the scrambled sentences task, the “Prime” and the “No-Prime” version, was used.

The Prime version consisted of 30 scrambled sentences [21] all including a word related to the concept of old age either directly translated from Bargh et al. [1] or adapted to French. To adapt the items, we conducted an online survey (80 participants) in which participants had to report 10 adjectives related to the concept of old age. Only the most frequent responses were used as replacement words. The word used as a prime could be put in a semantic context (e.g.: “always/worried/is/he/house/” for “he is always worried”) or not (e.g.: “piece/grey/of/Friday/fabric/a” for “a piece of grey fabric”). To complete the task, participants had to rearrange 30 scrambled sentences consisting of 4 to 5 words in the correct order and cross out the word that would not fit in the sentence (see supporting material S1 for a complete list of stimuli). There was no time limit to complete the task.

The No-Prime version of the task was exactly identical to the Prime version except that each word related to the concept of old age was replaced by a neutral word.

Participants were randomly and blindly assigned to either the Prime or the No-Prime condition. Experimenters were randomly recruited among students and had neither prior expectations toward participants behavior nor knowledge of the original experiment [1]. Each experimenter randomly tested participants from both conditions and was instructed to interact with each participant according to a strict script so that their potential influence was minimized. Experimenters remained in the room throughout the whole task. The questionnaires were enclosed in an envelope that the participant had to open, so as to keep each experimenter blind to the participant's condition. Upon completion of the scrambled sentences task, participants were thanked and dismissed. They then walked towards the exit through the hallway. In doing so, they triggered the infrared beams and their walking speed was subsequently computed.

Before reaching the exit, participants were called back by the experimenter who pretended that he/she had forgotten to administer a final task. The debriefing that followed relied on a funnel questionnaire [1], [18] assessing participants' awareness of the manipulation on three levels:

1-Awareness of the prime was assessed by asking participants increasingly specific questions about the presence of primes in the scrambled sentences. One particular question was a four-alternative forced-choice task (4-AFC) in which participants were required to choose between four pictures representing four social categories that could have been used as primes (i.e.: athletic person, Arabic person, handicapped person and elderly).
2-Awareness of the primed behavior was assessed by inviting participants to indicate how much they thought their walking speed had increased or decreased relative to their regular walking speed (responses were provided using an on-screen slider along a scale ranging from 0 to 100, with 50 representing their regular walking speed).
3-Awareness of the link between the prime and the primed behavior was assessed directly by asking participants whether they had noticed any link between the scrambled sentences task and their walking speed as they had left the room.

The debriefing was also used to probe suspicion regarding the purpose of the experiment by asking increasingly accurate questions such as: “Do you think this experiment is related to any topic in particular?”, “Do you think this experiment could be related with manipulating behavior?”.

### Results

#### Walking speed

In this analysis, we used participants' walking speed as they entered the experiment room, (i.e., before priming) as a covariate. The results show no significant difference between the Prime (M = 6.27″ SD = 2.15) and the No-Prime group (M = 6.39″ SD = 1.11) in the time necessary to walk along the hallway after the priming manipulation (F (1, 119)<1, η^2^ = .01).

#### Awareness of the prime

No participant reported having noticed anything unusual about the scrambled sentences task. Four participants (6.66%) in the Prime condition reported that the sentences were related to the stereotype of old persons. We tested the distribution of forced choices for both conditions using a two independent sample chi-squared test: the Prime group chose the picture of the old person above chance level whereas the No prime group was equally likely to choose all four pictures (χ2 (1) = 5.43, p = 0.023).

#### Awareness of the effect

We computed the deviation of the slider from the initial position. No significant difference was found between the Prime (M = 1.7) and the No-Prime (M = 2.68) groups (t (1, 119)<1, d = 0.022).

#### Awareness of the link

96% of participants reported that they could not establish a link between the scrambled sentences task and their subsequent behavior.

No experimenter reported having entertained any specific expectation about participants' behavior.

### Discussion

Despite our large sample of 120 participants (vs. 60 in Bargh et al.'s study 2a and 2b combined), and despite our precise measurement methods, we were unable to replicate the effects observed by Bargh et al. [1]. Further, and again in contrast to Bargh et al., the 4-AFC task we used revealed that participants showed a certain degree of awareness of the prime.

Based on these negative results, we decided to push the investigation further and elucidate the factors that could potentially promote Bargh et al's [1] effect on walking speed. A first possibility is simply that the effects observed by Bargh et al. results from a methodological artifact originating from the lack of accuracy of their time measurement method. There is also a second, more intriguing possibility. Studies [22] on automatic behavior have suggested that participants tend to mimic the experimenter's behavior. Therefore, it seemed plausible that experimenters' expectations about primed participants' behavior may have altered their own behavior (e.g.: slower interaction pace with the participants and gesture) and that these changes were communicated unconsciously by experimenters to the participants. Thus, an alternative explanation for Bargh et al's findings could be that participants adapt their behavior to their experimenters' expectations and hence walk slower as a result of a self-fulfilling prophecy [14].

Thus, as proposed by Dehaene et al's [9] framework, experimenters' expectations could act as an amplifier of the effect of the prime and thus promote the primed behavior.

To test these possibilities, we conducted a second experiment in which we manipulated the experimenters' expectations about primed participants' behavior. To assess the possible effects of the chronometry method, we also asked our experimenters to use a stopwatch in addition to the infrared sensors. .

## Experiment 2

### Methods

#### Participants

50 new participants (age range 18–30 years, average 21.3) took part in Experiment 2. 10 participants (age range 20–24 year, average 21.7) were also recruited as experimenters. This experiment was approved by the Ethics Committee of the Department of Psychological Sciences of the University of Brussels.

#### Procedure and design

In Experiment 2, we used the exact same design and procedure as in Experiment 1. However, two fundamental modifications were implemented.

#### Experimenters' expectations

Experimenters' expectations about primed participants' behavior were manipulated. One half of the experimenters were told that the primed participant would walk slower as result of the prime (i.e.: “Slow” condition), the other half were told that the participants would walk faster (i.e.: “Fast” condition). Each individual experimenter tested 5 participants randomly assigned to the Prime or the No-Prime condition. Experimenters' expectations were shaped through a one hour briefing and persuasion session prior to the first participant's session. In addition, the first participant whom an experimenter tested was a confederate who had been covertly instructed to act in the manner expected expected by the experimenter. Crucially, participants' condition (i.e.: Prime or No-Prime) was made salient to the experimenter. As in Experiment 1, the experimenters were instructed to follow a strict script to standardize their interactions with the participants and not to reveal to the participant the expected result of the prime on their subsequent behavior.

Experimenters' beliefs about the experimental set up and effect of the prime on the participants were assessed upon completion of their testing session.

#### Timings

As in Experiment 1, we used the infrared gate to measure participants' walking speed (hereafter, “objective timing”). However, the equipment was presented to the experimenters as experimental hardware that was so far unreliable and that needed further calibration. Therefore, we also asked the experimenters to measure participants' walking time using a manual stopwatch (hereafter, “subjective timing”).

### Results

To examine possible dependencies between observations due to the experimenter, we computed the intraclass coefficient for each of the key dependent variables. As this coefficient was always very low and not significantly different from 0 (p>.30), all observations were treated as independent [23].

In subsequent analyses, we distinguish three components in the timings. The subjective timings reflect the behavior of the experimenter measuring participants' walking speed. The objective data provided by the infrared sensors is a pure measure of participants' walking speed. The absolute difference between the subjective and the objective data is the absolute error and constitutes a measure of experimenters' accuracy.

**Figure 1:**
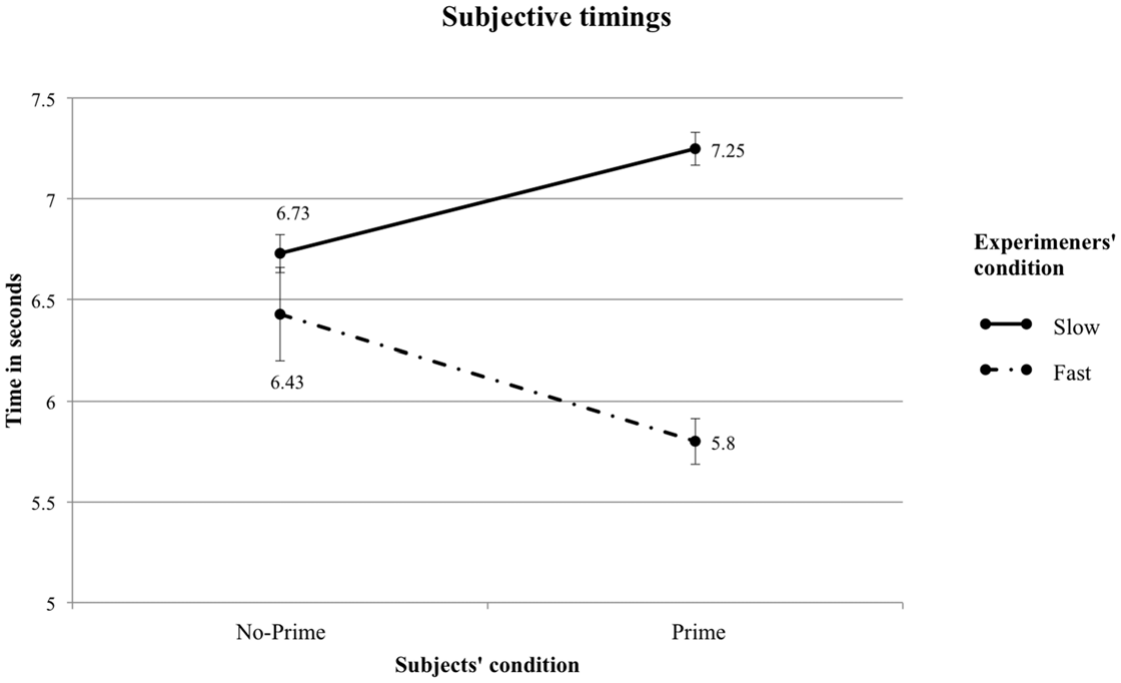
Subjective timings. Mean time in seconds to walk down the hallway measured manually by the experimenter. The error bars represent the standard error of the mean.

Concerning subjective timing data, Figure 1 shows that for subjective timings, participants in the Prime (M = 7.25″ SD = .33) condition walked significantly slower than those in the No-Prime (M = 6.73″ SD = .32) condition when tested by an experimenter expecting the primed participants to be slower (F(1, 24) = 12,32, p = .002, η^2^ = .339). This result replicates Bargh et al. [1] effect on walking speed. Strikingly however, as indicated by the significant interaction between participants' condition×experimenters' condition (F(3, 46) = 18,82, p<.001, η^2^ = .295), the effect was reversed in the Fast experimenters condition, so that Primed participants (M = 5.8″ SD = .73) walked faster than No-Prime participants (M = 6,43″ SD = .4) (F(1, 24) = 7,55, p = .012, η^2^ = .274).

**Figure 2:**
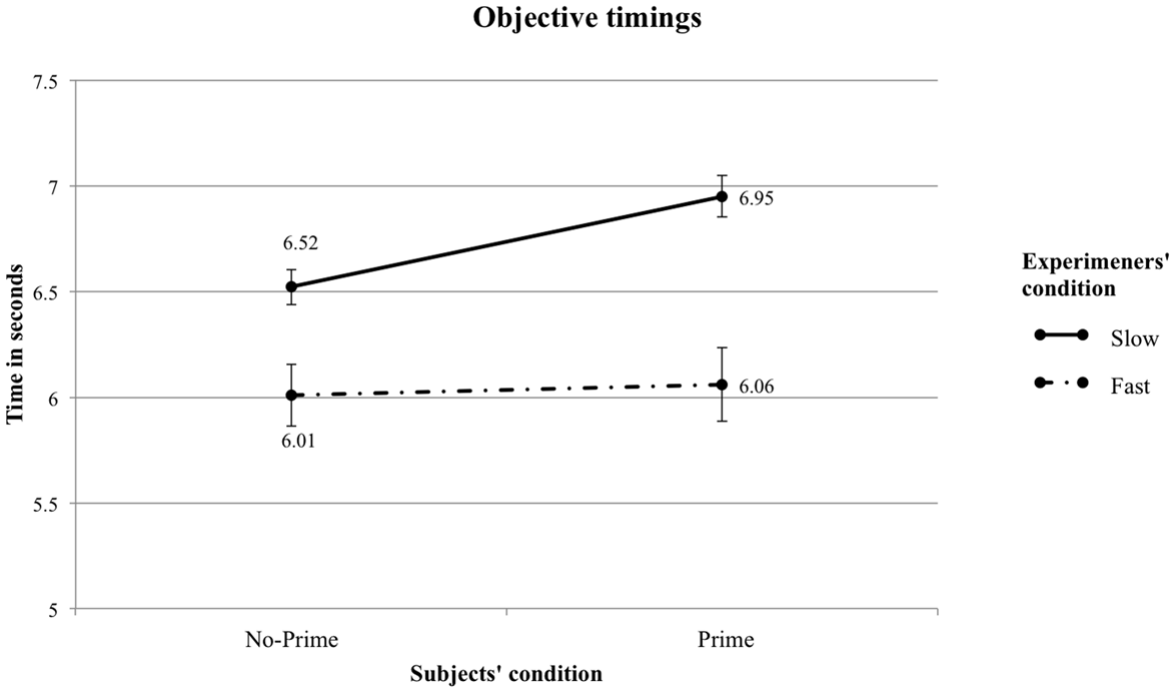
Objective timings. Mean time in seconds to walk down the hallway measured by the infrared sensors. The error bars represent the standard error of the mean.

In terms of objective timing (see Figure 2), we observed a difference between the Prime (M = 6.95″ SD = .36) and the No-Prime group (M = 6.52″ SD = .31) remains for the slow experimenter condition (F(1, 24) = 7.07, p = .014, η^2^ = .228). Bargh et al's [1] effect is thus replicated in this condition. There was no difference between the Prime (M = 6.07″ SD = .57) and the No-Prime (M = 6.01″ SD = .39) group (F(1, 24) = .231, p = .636, η^2^ = .011) in the fast experimenter condition. Finally, we also found a main effect of the experimenters' condition was found: participants tested by experimenters in the Slow condition (M = 6.75″ SD = .43) were slower than those tested by experimenters in the Fast condition (M = 6.04″ SD = .47) (F(1, 49) = 30.44, p<.001, η^2^ = .404).

**Figure 3:**
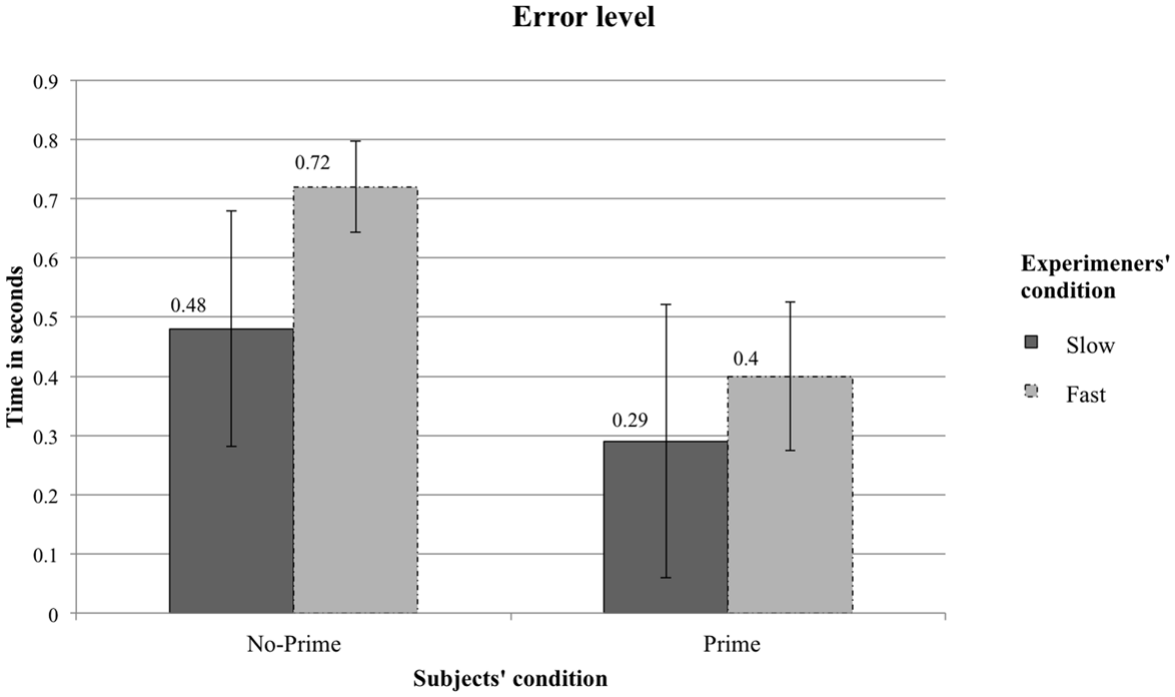
Error level. Absolute difference in seconds between the subjective and the objective timings. The error bars represent the standard error of the mean.

Turning now to the differences between objective and subjective timing data, which reflect experimenters' accuracy, Figure 3 shows that the reverse effect on walking speed for the Fast experimenter condition is explained by the absolute error: Experimenters in the Fast condition are less accurate for the No-Prime group (M = .41″ SD = .24) than for the Prime group (M = .73″ SD = .38) (F(1, 24) = 5.819, p = .023, η^2^ = .183). No significant difference was found between the Prime (M = 0.3″ SD = .17) and the No-Prime (M = 0.49″ SD = .32) groups (F(1, 24) = 3.297, p = .08, η^2^ = .113) in the Slow condition. Overall, the error level is the same for all groups, but for the No-Prime group in the Fast condition (F(49, 1) = 9.094, p = .004, η^2^ = .144).

#### Awareness of the prime

As in Experiment 1, analysis of responses to the forced choice questionnaire shows that the Prime group, over both experimenters' conditions, chooses the picture of the old person above chance level. In contrast, the No-prime group is equally likely to choose any of the four pictures (χ2 (1) = 5.62, p = 0.019).

#### Awareness of the effect

A significant difference was found between the Prime (M of the deviation = −7.3 SD = 1.35) and the No-Prime (M of the deviation = 2.01 SD = .87) group (F(1, 24) = 12.43, p = .042, η^2^ = .143) in the Slow experimenters' condition. No significant difference was found between the Prime (M of the deviation = 2.23 SD = .93) and the No-Prime group (M of the deviation = 3.12 SD = 1.05) in the Fast experimenters' condition (F(1, 24) = .56, p = .38, η^2^ = .015).

#### Awareness of the link

When asked if a link between the scrambled sentences task and their subsequent behavior could be made, 95% of participants in the Prime condition answered no. When assessed at the end of the experiment, every experimenter reported a high degree of belief in the cover story they were told in the pre-experimental briefing.

### Discussion

Can Bargh et al.'s [1] striking finding that walking speed can be influenced by unconscious primes associated with the stereotype of “old age” be replicated? Experiment 1, in which we used better timing methods, yielded null results. In Experiment 2, however, we were indeed able to replicate Bargh et al.'s priming effect, but only when the experimenter expected the primed participant to walk slower after having being primed. We observed the effect regardless of whether the chronometry method was objective or subjective. Strikingly, we were also able to reverse this effect in the subjective timings when the experimenters expected the primed participants to walk faster. Thus, if our experiment had used subjective timings only, as in Bargh et al's original study, we would have erroneously concluded that experimenters' expectations was a critical factor for the priming effect to occur. But the objective timings show otherwise. The reverse priming effect on walking speed observed on subjective timings stems from errors committed while operating the stopwatch: Experimenters in the fast condition were prone to commit more errors because they expected the need to capture a fast event and hence tended to be inaccurate in stopping the watch.

As in Experiment 1 we observed the same pattern of result regarding the awareness test. For the most part, primed participants were able to recall the concept related to the scrambled sentences task. Interestingly, primed participants in the Slow condition (i.e. those who actually walked slower as a result of the experimental manipulation) were to a certain degree aware of their diminished walking speed.

## Discussion

Our findings lead us to reconsider the results of Bargh et al's [1] experiments 2a and 2b with a critical eye and to consider their implications for nonconscious behavioral priming;

First, in Experiment 1, despite the use of a larger sample and an experimental procedure devoid of the limitations present in the original experiment, we were not able to replicate Bargh et al's [1] automatic effect of priming on walking speed. This led us to assume that crucial factors in this paradigm had remained unidentified. Experiment 2 was aimed at exploring such factors.

Second, in Experiment 2 we were indeed able to obtain the priming effect on walking speed for both subjective and objective timings. Crucially however, this was only possible by manipulating experimenters' expectations in such a way that they would expect primed participants to walk slower. Our results, however, cannot be explained solely in terms of a pure self-fulfilling prophecy effect [14], as the primed participants did not walk faster when tested by an experimenter who believed they would walk faster. Therefore it seems that the primes alone are not sufficient and must be in line with environmental cues such as the experimenters' behavior in order to elicit the effect on walking speed. This is also supported by the fact that contrary factors (i.e.: primes related to the concept of age in conjunction with an experimenter expecting to observer a faster walking speed) did not alter participants' walking speed.

Regarding the subjective timings, we obtained a reverse effect on walking speed (i.e.: participants walking faster). This effect can be explained by the error committed by the experimenters, most likely as a result of their induced expectations. If we had used only human-operated measurement devices, as in Bargh et al.'s [1] experiment 2a and 2b, we would thus have erroneously concluded in a reverse priming effect. This very important point suggests that one must be cautious about (1) the type of measurement used in behavioral priming experiments as well as (2) the experimenters expectations. Subtle differences are particularly prone to external influences and potential biases.

Third, most of the participants primed with the scrambled sentences task were aware of the social category they had been primed with. This result shows that one must be cautious when using the scrambled sentences task as a priming method. One must take into account that our participants were performing the experiment as part of a psychology course, which could have led to higher suspicion towards the scrambled sentences task resulting in a higher degree of awareness of the primes. Additionally, those participants who actually exhibited a slower walking speed reported in good proportion being aware of that particular behavior. Whether automatic behavioral priming can occur without awareness thus remains unclear. As a matter of fact, participants' awareness of the prime and of the primed behavior could have led them to exert better conscious control over the latter and therefore impair its expression.

Finally, priming alone was not sufficient to promote a priming effect on walking speed comparable to Bargh et al's [1]. We also had to manipulate experimenters' beliefs so that they would expect the primed subjects to walk slower. This finding is congruent with recent evidence showing that primed behavior is sensitive to the context in which it takes place [24], [25]. The present finding can be seen in the same light. Experimenters' expectations seem to provide a favorable context to the behavioral expression of the prime. Obviously, this interpretation remains tentative, as we do not know how this process operates. However, it is likely that experimenters who expect their participants to walk slower behave differently than those who expect their participants to walk faster and that such behavioral cues are picked up by participants.

In conclusion, although automatic behavioral priming seems well established in the social cognition literature, it seems important to consider its limitations. In line with our result it seems that these methods need to be taken as an object of research per se before using it can be considered as an established phenomenon.

## Supporting Information

Supporting Material S1 **list of stimuli.** Here is the list of the primes and neutral words as well as their English translation used in both Experiment 1 and 2.
(DOCX)
