# Supplementary material for: Behavioral Priming: It's All in the Mind, but Whose Mind?
Source: PLoS One. 2012 Jan 18;7(1):e29081. doi: 10.1371/journal.pone.0029081 (PMC3261136; doi:10.1371/journal.pone.0029081)
Supplement: Supporting Material S1 — list of stimuli. Here is the list of the primes and neutral words as well as their English translation used in both Experiment 1 and 2. (DOCX) [file pone.0029081.s001.docx]

**Supporting material S1**

| **Prime words**  **(Fr)** | **Prime words**  **(Eng)** | **Neutral words**  **(Fr)** | **Neutral words**  **(Eng)** |
| --- | --- | --- | --- |
| \| Inquiet \| \| --- \| \| Vieil \| \| Solitaire \| \| Gris \| \| Egoïstement \| \| Prudente \| \| Sentimental \| \| Sages \| \| Têtu \| \| Courtois \| \| Isolé \| \| Oublieux \| \| Retraité \| \| Ridée \| \| Rigides \| \| Traditionnel \| \| Amère \| \| Conservatrice \| \| Tricot \| \| Dépendant \| \| Anciens \| \| Impuissant \| \| Crédule \| \| Précaution \| \| Seuls \| \| Canne \| \| Malade \| \| Repos \| \| Blanche \| \| Home \| | \| Worried \| \| --- \| \| Old \| \| Lonely \| \| Grey \| \| Selfishly \| \| Careful \| \| Sentimental \| \| Wise \| \| Stubborn \| \| Courteous \| \| Isolated \| \| Forgetful \| \| Retired \| \| Wrinkled \| \| Rigid \| \| Traditional \| \| Bitter \| \| Conservative \| \| Knit \| \| Relying \| \| Ancient \| \| Powerless \| \| Gullible \| \| Precautious \| \| Alone \| \| Cane \| \| Ill \| \| Rest \| \| White \| \| Retirement house \| | \| Présent \| \| --- \| \| Petit \| \| Affamé \| \| Blond \| \| Généreusement \| \| Rigoureux \| \| Nerveux \| \| Bon \| \| Chargé \| \| Ample \| \| Brun \| \| Déguisé \| \| Différent \| \| Jaune \| \| Connues \| \| Chaud \| \| Verte \| \| Empiriques \| \| Ricochet \| \| Grand \| \| Juristes \| \| Perplexe \| \| Occupé \| \| Maitrise \| \| Arrivés \| \| Statuette \| \| Skieurs \| \| Repas \| \| Rouge \| \| Bateau \| | \| Present \| \| --- \| \| Small \| \| Starved \| \| Blond \| \| Generously \| \| Rigorous \| \| Nervous \| \| Good \| \| Loaded \| \| Ample \| \| Brown \| \| Disguised \| \| Different \| \| Yellow \| \| Known \| \| Warm \| \| Green \| \| Empirical \| \| Rebound \| \| Tall \| \| Jurist \| \| Perplex \| \| Busy \| \| Maitrise \| \| Arrived \| \| Statuette \| \| Skiers \| \| Meal \| \| Red \| \| Boat \| |
